# Human pharmacokinetics of XBD173 and etifoxine distinguish their potential for pharmacodynamic effects mediated by translocator protein

**DOI:** 10.1111/bcp.15392

**Published:** 2022-05-20

**Authors:** David R. Owen, Alexandra Phillips, Desmond O’Connor, Gabrielle Grey, Lina Aimola, Richard Nicholas, Paul M. Matthews

**Affiliations:** ^1^ Department of Brain Sciences Imperial College London London; ^2^ Pharmidex Pharmaceutical Services London UK; ^3^ UK Dementia Research Institute Centre Imperial College London London UK

**Keywords:** anti‐inflammatory drugs, brain, etifoxine, translocator protein, XBD173

## Abstract

XBD173 and etifoxine are translocator protein (TSPO) ligands that modulate inflammatory responses in preclinical models. Limited human pharmacokinetic data is available for either molecule, and the binding affinity of etifoxine for human TSPO is unknown. To allow for design of human challenge experiments, we derived pharmacokinetic data for orally administered etifoxine (50 mg 3 times daily) and XBD173 (90 mg once daily) and determined the binding affinity of etifoxine for TSPO. For XBD173, maximum plasma concentration and free fraction measurements predicted a maximal free concentration of 1.0 nM, which is similar to XBD173 binding affinity. For etifoxine, maximum plasma concentration and free fraction measurements predicted a maximal free concentration of 0.31 nM, substantially lower than the *K*_*i*_ for etifoxine in human brain derived here (7.8 μM, 95% CI 4.5–14.6 μM). We conclude that oral XBD173 dosing at 90 mg once daily will achieve pharmacologically relevant TSPO occupancy. However, the occupancy is too low for TSPO mediated effects after oral dosing of etifoxine at 50 mg 3 times daily.

**What is already known about this subject**

- Pharmacological and genetic modulation of the translocator protein (TSPO) is immunomodulatory and biases microglia towards expression of immunosuppressive phenotypes. XBD173 and etifoxine are TSPO ligands that have been explored extensively in preclinical models and are now being used in human challenge experiments.
- There are limited data available on plasma concentrations and plasma free fraction following administration to humans, and on the TSPO binding affinity of etifoxine. These are required to best design these experiments.

**What does this study add**

- The pharmacokinetic and brain‐binding affinity data provided here are consistent with potential anti‐inflammatory activity of orally administered XBD173 that is mediated by TSPO.
- The free plasma concentration of etifoxine is too low for pharmacologically relevant TSPO occupancy by etifoxine at the standard clinical dose

## 1 INTRODUCTION

Pharmacological and genetic modulation of the translocator protein (TSPO) is immunomodulatory and biases microglia towards expression of immunosuppressive phenotypes in vitro.^1^, ^2^, ^3^, ^4^, ^5^, ^6^ Preclinical in vivo evidence for an anti‐inflammatory effect of TSPO ligands also is compelling; in a range of neurodegenerative and inflammatory mouse models, TSPO ligands inhibit proinflammatory activation and improve clinical scores.^1^, ^6^, ^7^, ^8^, ^9^, ^10^, ^11^, ^12^, ^13^, ^14^, ^15^ These experiments suggest that TSPO may be a novel target for central nervous systems disorders which are partly driven by neuroinflammatory mechanisms. TSPO ligands have also been investigated as tools to enhance neurosteroid synthesis and hence as potential treatments for disorders driven partly by reduced neurosteroid concentrations.^16^

XBD173 and etifoxine are TSPO ligands that have been explored extensively in preclinical models^14^, ^17^, ^18^, ^19^, ^20^, ^21^, ^22^, ^23^, ^24^, ^25^, ^26^, ^27^, ^28^ and clinical studies.^16^ Etifoxine is licensed for the treatment of anxiety in France. XBD173 has previously been administered to humans in an experimental medicine study.^16^ However, while both have been believed to be acting through modulation of TSPO, differences in pharmacodynamics have been reported. For example, etifoxine improved clinical scores in an experimental autoimmune encephalomyelitis mouse model, whereas XBD173 did not.^20^

Here we sought to gather appropriate pharmacokinetic and binding affinity data to enable use of these molecules in challenge studies in humans for exploration of effects of TSPO modulation. Although etifoxine is used clinically, there are no publicly available pharmacokinetic data. The binding affinity of etifoxine for TSPO in the rodent brain is approximately 12 μM,^29^ but the affinity in the human brain has not been reported publicly. The influence of differences in TSPO structure with the common rs6971 polymorphism, which changes the affinity of TSPO for many ligands,^30^ is also unknown. To test whether the current clinically approved dosing schedule of etifoxine produces plasma concentrations which are consistent with occupancy of TSPO, we measured etifoxine binding affinity for TSPO in the human brain in subjects having different rs6971 genotypes and performed pharmacokinetic studies at the approved clinical dose of 50 mg 3 times daily (TDS) in healthy volunteers.

XBD173 was developed for the treatment of anxiety disorders. Pharmacokinetic studies with XBD173 suggested that a 90‐mg dose produces peak plasma concentrations in the micromolar range.^16^ However, positron emission tomography (PET) data subsequently showed only 80% TSPO peak occupancy in people with the rs6971 common variant (*high affinity binders*, HAB)^30^ following a 90‐mg dose.^31^ This occupancy figure is substantially lower than would be predicted, as the affinity of XBD173 for TSPO in the human brain is 2–3 nM and hence micromolar concentrations would be expected to saturate it.^32^ To better understand this dose occupancy relationship, we repeated pharmacokinetic analyses of XBD173 in healthy volunteers and conducted measurements of the plasma‐free fraction.

## 2 METHODS

### 2.1 Clinical study design

This was a single centre, open‐label study enrolling 4 adult healthy volunteers (aged 35–65 y) irrespective of rs6971 genotype. Detailed inclusion and exclusion criteria are shown in the supplementary information. Eligible participants were randomised via an online system hosted by a specialist company (www.sealedenevelope.com) to receive either once daily oral XBD173 (90 mg) continuously for 7 days, a minimum 28‐day washout, and then 3 times daily oral etifoxine (50 mg) continuously for 7 days, or vice versa. Although both drugs have short plasma half‐lives, the kinetics of the biological effects are unknown. As a precaution, therefore, a long washout period was chosen. The first dose was taken at the clinical research facility and the participant was monitored for 4 hours. Subsequent doses were taken at home by the participant. On day 7, the participant returned for a final assessment. The study protocol was approved by West London & GTAC Research Ethics Committee (ref 17/LO/0566), and all participants provided written informed consent. The PI of the study was Paul M. Matthews (DPhil, FRCP, FMedSci) who is an author on the manuscript.

### 2.2 Pharmacokinetics

Plasma samples for pharmacokinetic analysis were obtained on day 1 at 0.5, 1, 2, 3, 4 hours following the first dose. As there are no data on whether etifoxine accumulates following repeated dosing, a further sample was taken on the final day of etifoxine dosing, approximately 2–3 hours following the final dose of the drug. This analysis was not performed for XBD173 as this molecule does not accumulate.^16^ Plasma samples were stored at −20°C or lower until analysis. Plasma samples (25 μL) were prepared for analysis by protein precipitation with acetonitrile containing internal standard (tolbutamide; 200 μL) followed by mixing (150 rpm, 15 min) and centrifugation (1500 g, 15 min). The supernatant (50) μL was diluted with water (100 μL) and mixed (100 rpm, 15 min). XBD173 samples were analysed by liquid chromatography–tandem mass spectrometry (LC–MSMS) (Shimadzu Nexera X2 UHPLC/Shimadzu LCMS 8060) with Phenomenex Kinetex Biphenyl (50 × 2.1 mm), 1.7‐μm column and mobile phase components water/0.1% formic acid (A) and acetonitrile/0.1% formic acid (B). Mobile phase gradient was 0–0.3 minutes 2% B; 0.3–1.1 minutes increase to 95% B; 1.1–1.75 minutes 95% B, 1.75–1.8 minutes decrease to 2% B; 1.8–2.5 minutes 2% B. Flow rate was 0.4 mL/min. Injection volume was 1 μL. Etifoxine was analysed in the same way except column was Waters Aquity BEH C18 (50 × 2.1 mm), 1.7 μm. MSMS transitions for XBD173, etifoxine and tolbutamide were 401.9 > 227.1, 300.9 > 230.1 and 271.0 > 91.0 respectively. Calibration standards were prepared by spiking XDB173 or etifoxine into control plasma over the ranges 2–10 000 ng/mL (XDB173) or 1–5000 ng/mL (etifoxine), then preparing and analysing as for the study samples. Lower limit of detection was 2 ng/mL for XDB173 and 1 ng/mL for etifoxine.

### 2.3 Plasma protein binding determination

Plasma protein binding was measured by equilibrium dialysis using 96‐well plate blocks with 2 compartments separated by a vertical cylinder of dialysis membrane (molecular weight cut off ~8000 Da; ThermoFisher Scientific). Plasma spiked with either etifoxine or XBD173 at 1 μM final concentration was placed in the first compartment, in the other was placed phosphate buffered saline, pH 7.4. The unit was covered and incubated at 37°C on an orbital shaker for 4 hours. After incubation, samples from both plasma (bound) chamber and the buffer (free) chamber were taken and matrix‐matched by addition of the alternative blank matrix. Acetonitrile containing internal standard was added to all samples. The samples were centrifuged, and the supernatant was analysed by LC–MS (Agilent 1290 UHPLC/Agilent 6550 QToF/Waters Acquity BEH Phenyl (50 x 2.1 mm) 1.7‐μm column/0.1% formic acid, acetonitrile 0.1% formic acid mobile phase gradient with flow 0.4 mL/min). The ratio of responses in free to bound samples equates to the free fraction.

### 2.4 Materials

XBD173 was manufactured according to good manufacturing practice as a custom preparation by Pharmasynth, Estonia. Etifoxine was obtained from the hospital pharmacy. [^3^H]PK11195(1‐(2‐Chlorophenyl)‐*N*‐methyl‐*N*‐(1‐methylpropyl)‐3‐isoquinolinecarboxamide specific activity = 80 Ci/mmol; radioactive concentration = 1.0 mCi/mL) was purchased from Perkin Elmer, UK. Unlabelled PK11195 and etifoxine was obtained from Sigma, UK. Nomenclature related to drugs and molecular targets conforms to the IUPHAR/BPS Guide.^33^

### 2.5 Radioligand binding experiments

Details of the radioligand binding experiments are in the supplementary methods. In brief, brain tissue was obtained from 4 HABs and 4 low affinity binders (LABs) from the UK Multiple Sclerosis Tissue Bank at Imperial College and stored at −80°C until use. Binding affinity status was determined previously by radioligand binding experiments using [3H]PK11195 and unlabelled PBR28.^34^ Aliquots of membrane suspension were incubated with [3H]PK11195 (0.3 nM) and 1 of 8 concentrations of etifoxine ranging from 150 nM to 30 μM. The assay was terminated via filtration through Whatman GF/B filters. Scintillation fluid (4 mL/vial, Perkin Elmer Ultima‐Gold MV) was added and vials counted on a Perkin Elmer Tricarb 2900 liquid scintillation counter. A dissociation constant for [3H]PK11195 of 29.25 nM^6^ was used to generate the inhibition constant (*K*i) for etifoxine according to the Cheng and Prusoff Equation^12^ using GraphPad Prism 8.1 software (GraphPad Software Inc, USA).

## 3 RESULTS

### 3.1 Participant characteristics

Four participants (1 female, 3 male) with mean age 51.5 years were separately dosed with XBD173 and etifoxine. Three participants were HABs and 1 was a mixed affinity binder. The washout period in between the dosing periods ranged from 28 to 42 days. All participants completed all visits. No adverse events were reported.

### 3.2 Pharmacokinetics

Following oral administration of 90 mg XBD173, the median plasma maximum plasma concentration (Cmax) and time to Cmax were 114 ng/mL and 2.5 hours, respectively (Table 1). Following oral administration of 50 mg etifoxine, the median plasma Cmax and time to Cmax were 32 ng/mL and 2.0 hours, respectively. We did not find evidence for significant etifoxine accumulation after multiple doses: plasma etifoxine concentrations following the final dose on the morning of day 7 were not higher than on day 1 (Supplementary Table 2).

**TABLE 1:** Pharmacokinetic parameters following oral administration of a single dose of 90 mg XBD173 or 50 mg etifoxine

	Participant number						
	1	2	3	4	Min	Max	Median
90 mg XBD173 single dose							
Tmax (h)	2.0	4.0	2.0	3.0	2	4	2.5
Cmax (ng/mL)	56	25	263	171	25	263	114
AUClast (ng h/mL)	148	74	556	345	74	556	247
50 mg Etifoxine single dose							
Tmax (h)	3.0	2.0	2.0	2.0	2	3	2.0
Cmax (ng/mL)	24	33	39	31	24	39	32
AUC_0‐4 h_ (ng h/mL)	59	64	83	71	59	83	68

AUC, area under the time–plasma concentration curve; Cmax, maximum plasma concentration; Tmax, time to Cmax.

### 3.3 Plasma free fraction

We assessed plasma free fractions using equilibrium dialysis and found that 99.66% (standard error of the mean, SEM 0.03) of the XBD173 in plasma was bound and 0.34% (SEM 0.03) free and that 99.71% (SEM 0.02) of the etifoxine was bound and 0.29% (SEM 0.02) free.

### 3.4 Estimation of etifoxine *K*_i_ in brain tissue

**FIGURE 1:**
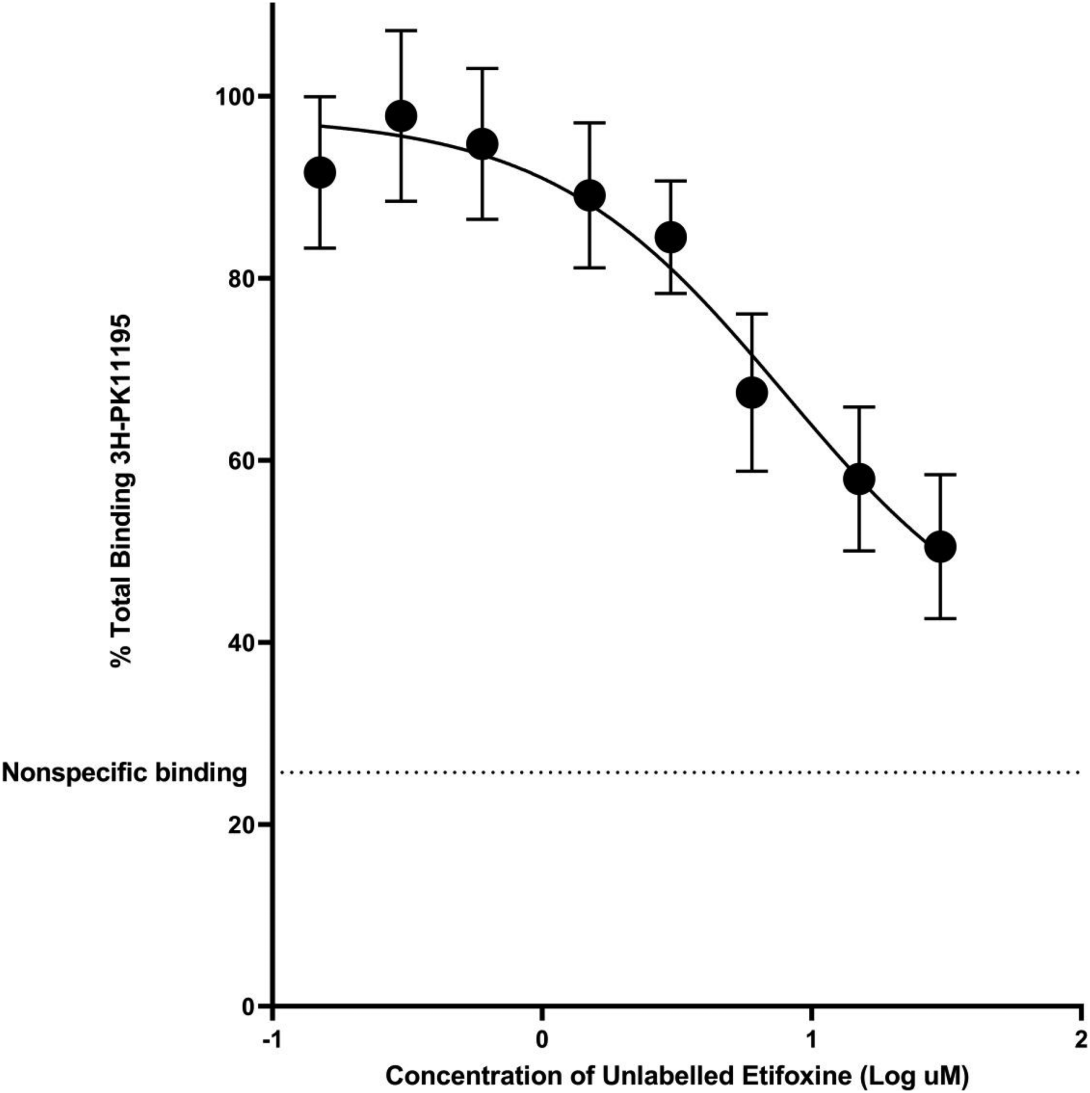
Competition assay with [^3^H]PK11195 and unlabelled etifoxine, using human brain tissue. Each data point represents the mean value of all subjects, and the error bars represent standard error of the mean. Dotted line represents nonspecific binding determined by unlabelled PK11195 (10 μM). Data from high affinity and low affinity binders are plotted in 1 curve as etifoxine affinity was independent of rs6971 genotype.

Competition assays with unlabelled etifoxine were performed with brain tissue from 8 donors (4 HABs, 4 LABs). The mean *K*_*i*_ value for the HABs (7.6 ± 2.2 μM, *n* = 4) was similar to that of the LABs (7.6 ± 1.7 μM, *n* = 4; *P* = .99). The estimated *K*_*i*_ value for the whole population when fitted to a single site binding curve was 7.8 μM (95% CI 4.5–14.6 μM; Figure 1). Due to the limitation on etifoxine solubility, the competition curve did not fully plateau.

### 3.5 Estimates of TSPO occupancy

The median Cmax of 114 ng/mL and free fraction of 0.34%, suggests a predicted free plasma XBD173 concentration of approximately 1.0 nM and a TSPO occupancy of ~30% in HABs.

For etifoxine, with an average Cmax was 32 ng/mL and free fraction of 0.29%, the predicted free etifoxine concentration was estimated to be approximately 0.31 nM. Given the affinity of etifoxine for TSPO estimated here (7.6 μM), this implies that TSPO occupancy of etifoxine at peak concentration will be <0.01%.

## 4 DISCUSSION

XBD173 and etifoxine have been explored as possible immunomodulatory TSPO ligands in preclinical and in vitro models. Here we sought to gather appropriate pharmacokinetic and binding affinity data to enable design of TSPO challenge studies in humans for future pharmacodynamic investigations.

We found that the affinity of etifoxine for TSPO in the human brain is approximately 7.8 μM, irrespective of rs6971 genotype. This estimate is similar to measures previously reported for the rodent brain (12.5 μM), heart (22.5 μM) and kidney (14 and 9 μM).^29^, ^35^, ^36^ However, following a 50‐mg oral dose, we also estimated a sub‐nanomolar free plasma concentration from our pharmacokinetic studies. Assuming equilibrium between free plasma concentration and brain tissue concentrations, this would equate to TSPO occupancy of approximately 0.01%. We also did not find evidence for drug accumulation after dosing at 50 mg TDS for 7 days. Indeed, plasma etifoxine concentration on day 7 was lower than on day 1, which may reflect variability or that a participant took the day 7 morning dose later than directed. We therefore conclude that any pharmacodynamic effects of etifoxine with a dose regimen of 50 mg TDS are not mediated by its interaction with TSPO. These low free plasma concentrations of etifoxine also appear inconsistent with pharmacological activity at the γ‐aminobutyric acid‐a receptor, the presumed target for anxiety.^37^, ^38^ Although the affinity has not been estimated in human tissue, in the rodent brain, etifoxine binds the γ‐aminobutyric acid‐a receptor with an IC50 of approximately 6.7 μM, and pharmacodynamic actions at the receptor are elicited only above 1 μM.^35^ If plasma free concentration reflects concentration in the brain, any pharmacodynamic effect due to etifoxine at the standard dose is likely to be independent of this target.

PET data with XBD173 showed that a 90‐mg dose is associated with only approximately 80% occupancy of TSPO in the brain,^31^ despite plasma concentrations reaching 1 μM.^16^ Our estimates of plasma concentration were lower (~320 nM) but would still predict saturation (>99% occupancy) of TSPO by XBD173. Here we also have shown that if free fraction is used for estimation of TSPO occupancy, assuming equilibrium between plasma concentration and brain tissue concentration, the previously observed in vivo TSPO occupancy can be predicted from XBD173 pharmacokinetic data. However, we also show there may be substantial intersubject variability in XBD173 plasma concentration, implying that 90 mg may be insufficient to achieve high TSPO occupancy in some participants.

Our study has important limitations. First, the sample size for the pharmacokinetic study was small. Second, we took only 5 samples over 4 hours and may therefore have underestimated Cmax. Third, due to limited solubility of etifoxine, the competition curve for the radioligand binding experiment with etifoxine did not plateau. The result of these limitations is that our estimates for the plasma concentrations and etifoxine binding affinity lack precision. However, the aim of this study was to determine whether the plasma concentrations were consistent with TSPO occupancy. The disparity between our estimates of etifoxine binding affinity and free plasma concentration is substantial. It is therefore unlikely that more precise estimates would materially alter the conclusions. When estimating TSPO occupancy of etifoxine in the brain, we made the assumption that free plasma and free brain concentrations of etifoxine are in equilibrium. This may not be the case. To definitively determine whether etifoxine binds TSPO in the brain at the administered doses, a PET occupancy study would be required. Finally, we did not formally assess compliance, beyond measuring etifoxine plasma concentration on day 7. It is therefore possible that day 7 etifoxine concentrations were no higher than day 1 concentrations because the participants did not take the drug as directed.

## 5 CONCLUSION

The pharmacokinetic and brain binding affinity data are consistent with potential anti‐inflammatory activity of orally administered XBD173 that is mediated by TSPO. However, the free plasma concentration of etifoxine is too low for pharmacologically relevant TSPO occupancy by etifoxine at the approved clinical dose of 50 mg TDS.

## COMPETING INTERESTS

P.M.M. notes consultancy fees from Novartis and Biogen. He has received honoraria or speakers' honoraria from Novartis, Biogen and Roche and has received research or educational funds from Biogen, Novartis and GlaxoSmithKline.

## CONTRIBUTORS

D.R.O. wrote manuscript, designed research, performed research, analysed data.

A.P. wrote manuscript, performed research, analysed data.

D.O.C. designed research, analysed data.

G.G. wrote manuscript, performed research.

L.A. wrote manuscript, performed research.

R.N. designed research, performed research.

P.M. wrote manuscript, designed research.

## Supporting information


**TABLE S1** Demographic, disease and tissue handling details.
**TABLE S2** Plasma concentrations following oral administration of 90 mg XBD173 or 50 mg etifoxine

## Data Availability

The data that support the findings of this study are available from the corresponding author upon reasonable request. Some data may not be made available because of privacy or ethical restrictions.
