# Supplementary material for: Human pharmacokinetics of XBD173 and etifoxine distinguish their potential for pharmacodynamic effects mediated by translocator protein
Source: Br J Clin Pharmacol. 2022 May 20;88(9):4230–6. doi: 10.1111/bcp.15392 (PMC9545781; doi:10.1111/bcp.15392)
Supplement: Supplementary file 1 — TABLE S1 Demographic, disease and tissue handling details. TABLE S2 Plasma concentrations following oral administration of 90 mg XBD173 or 50 mg etifoxine [file BCP-88-4230-s001.doc]

**Supplementary information**

Inclusion Criteria for PK studies

- Capable of giving written informed consent, which includes compliance with the requirements and restrictions listed in the consent form.
- Aged 35-65 years old
- A female subject is eligible to participate if she is a) of non-childbearing potential, defined as pre-menopausal females with a documented tubal ligation or hysterectomy, or postmenopausal defined as 12 months of spontaneous amenorrhea or b) of childbearing potential but not pregnant (as determined by urinary pregnancy test on screening and on each study day) and willing to use one of the contraception methods listed below
- Male subject must agree to use one of the contraception methods listed above.
- Willing to abstain from alcohol for the duration of dosing.

Exclusion Criteria for PK studies

- Clinically meaningful abnormalities in routine bloods including eGFR < 60ml/min, Elevation of liver enzymes/bilirubin, Prolonged prothrombin time, Thrombocytopenia
- Use of the following medications or therapies: Central nervous system depressants, P450 CY3A4 inducers or inhibitors, oral contraceptives, oral anticoagulants or antiplatelet agents other than low dose aspirin, levothyroxine
- Currently breastfeeding
- Any clinical significant medical conditions that in the opinion of the investigator would compromise subjects’ safety or compliance with study procedures.
- History of any clinical condition which in the opinion of the principal investigator would compromise the scientific integrity of the study, such as some chronic systemic diseases affecting blood, liver or kidneys or endocrine system
- Unwillingness or inability to follow the procedures outlined in the protocol
- Subject is mentally or legally incapacitated
- Contraindication to XBD173 use (Hypersensitivity to the active substance or to any of the excipients)
- Contraindication to etifoxine use (Myasthenia gravis, syndromes of glucose and galactose malabsorption or lactose deficiency)

***Methods for radioligand binding assays***

***Brain tissue***

Tissue was obtained from 8 donors from the UK Multiple Sclerosis (MS) Tissue Bank at Imperial College. Previous work has shown that TSPO binding affinity is no different in donors with MS versus those with no antemortem neurological diagnosis (1). The tissue was stored at -80^o^C until use. Demographic, tissue handling and clinical information concerning the donor is found in Supplementary Table 1. The binding profiles of these donors has been established previously using a competition assay with [^3^H]PK11195 and unlabelled PBR28: 4 donors were high affinity binders (HABs) and 4 were low affinity binders (LABs) (2).

***Membrane preparation***

Tissue blocks were homogenised in 10 times weight for volume (w/v) buffer (0.32mM sucrose, 5mM Tris base, 1mM MgCl_2_, pH 7.4, 4^o^C). Homogenates were centrifuged (32,000*g*, 20min, 4^o^C) followed by removal of the supernatant. Pellets were re-suspended in at least 10 times w/v buffer (50mM Tris base, 1mM MgCl_2_, pH 7.4, 4^o^C) followed by two washes by centrifugation (32,000*g*, 20min, 4^o^C). Membranes were suspended in buffer (50mM Tris base, 1mM MgCl_2_, pH 7.4, 4^o^C) at a protein concentration of approximately 4 mg protein/ml and aliquots were stored at -80^o^C until use.

***Competition binding assays***

Aliquots (approximately 250μg protein/ml) of membrane suspension were prepared using assay buffer (50mM Tris base, 140mM NaCl, 1.5mM MgCl_2_, 5mM KCl, 1.5mM CaCl_2_, pH 7.4, 37^o^C) and incubated with [^3^H]PK11195 (0.3nM) and one of 8 concentration of etifoxine ranging from 150nM to 30µM, in a final volume of 500µl for 60 min at room temperature. As has been previously been reported, we could not study concentrations higher than 30µM due to limitations of solubility (3). The specific binding component was determined using unlabelled PK11195 (10µM). Following incubation, assays were terminated via filtration through Whatman GF/B filters, followed by 3 x 1 ml washes with ice-cold wash buffer (50mM Tris Base, 1.4mM MgCl_2_, pH 7.4, 4^o^C). Whatman GF/B filters were pre-incubated with 0.05% polyethyleneimine (60min) before filtration. Scintillation fluid (4ml/vial, Perkin Elmer Ultima Gold MV) was added and vials counted on a Perkin Elmer Tricarb 2900 liquid scintillation counter. For each donor, each point was performed in quadruplicate. *K*_i_ (nM) values were determined using GraphPad Prism 8.1 software (GraphPad Software Inc, USA).

***Protein concentration determination***

Protein concentrations (μg protein/ml) were determined using the Bicinchoninic acid assay (BCA Kit, Sigma-Aldrich, UK) and absorption read at 562 nm.

***Data analysis***

All competition data were analysed using the iterative non-linear regression curve fitting software supplied with GraphPad Prism 8.1. A single site competition model was fitted to the data using the least squares algorithm. A *K*_d_ for [^3^H]PK11195 of 29.25nM *(6)* was used to generate the *K*_i_ for etifoxine according to the Cheng and Prusoff equation *(12)*. Data are expressed as the mean ± standard error of the mean (SEM). The Student’s t-test (GraphPad Prism 8.1) was used to determine statistical significance.

**References**

1. Owen DR, Gunn RN, Rabiner EA, Bennacef I, Fujita M, Kreisl WC, et al. Mixed-affinity binding in humans with 18-kDa translocator protein ligands. J Nucl Med. 2011;52(1):24-32.

2. Owen DR, Howell OW, Tang SP, Wells LA, Bennacef I, Bergstrom M, et al. Two binding sites for [3H]PBR28 in human brain: implications for TSPO PET imaging of neuroinflammation. J Cereb Blood Flow Metab. 2010;30(9):1608-18.

3. Schlichter R, Rybalchenko V, Poisbeau P, Verleye M, Gillardin J. Modulation of GABAergic synaptic transmission by the non-benzodiazepine anxiolytic etifoxine. Neuropharmacology. 2000;39(9):1523-35.

**Supplementary Table 1**

Demographic, disease and tissue handling details.

| Donor  Number | Sex | Age at  Death  (years) | Time from death  to organ  harvesting (hours) | Cause of death | Disease  Duration  (years) |
| --- | --- | --- | --- | --- | --- |
| **High affinity binders** |  |  |  |  |  |
| 1 | F | 42 | 31 | Multiple sclerosis | 20 |
| 2 | F | 59 | 21 | Bronchopneumonia | 39 |
| 3 | M | 66 | 16 | Gastrointestinal bleeding | 29 |
| 10 | F | 39 | 18 | Bronchopneumonia | 21 |
| **Low affinity binders** |  |  |  |  |  |
| 5 | F | 64 | 7 | Gastrointestinal bleed | 36 |
| 20 | F | 44 | 20 | Sepsis | 19 |
| 21 | M | 46 | 7 | Bronchopneumonia | 8 |
| 22 | F | 53 | 17 | Multiple sclerosis | 28 |

**Supplementary Table 2**

Plasma concentrations following oral administration of 90mg XBD173 or 50mg etifoxine

| **Timepoint**  (mins) | **Participant number** | | | | **Mean (SEM)** |
| --- | --- | --- | --- | --- | --- |
|  | 1 | 2 | 3 | 4 |  |
| 90mg XBD173 single dose | | | | | |
| 30 | ND | 5.7 | ND | ND | NA |
| 60 | 28.4 | 15.4 | 62.7 | 18.4 | **31.2 (10.9)** |
| 120 | 55.8 | 24.2 | 262.7 | 82.7 | **106.4 (53.5)** |
| 180 | 43.3 | 23 | 161.1 | 171.2 | **99.7 (38.7)** |
| 240 | 40.4 | 24.5 | 139.2 | 146.1 | **87.6 (32.0)** |
| 50mg Etifoxine single dose | | | | | |
| 30 | ND | 4 | ND | ND | NA |
| 60 | ND | 15.9 | 6.2 | 2.4 | **8.2 (4.0)** |
| 120 | 15.5 | 33.4 | 39.4 | 30.7 | **29.8 (5.1)** |
| 180 | 24.2 | 12.3 | 27.1 | 27.9 | **22.9 (3.6)** |
| 240 | 22.7 | 9.2 | 20.1 | 20.1 | **18.0 (3.0)** |
| Etifoxine multiple dosing (50mg TDS) | | | | | |
| Day 7 (2-3 hours post dose) | 14.0 | 23.5 | 11.3 | 3.7 | **13.1 (4.1)** |

Figures quoted are ng/mL

Abbreviations: ND; Not detected. NA; Not applicable
